# A Visual Two-Choice Rule-Switch Task for Head-Fixed Mice

**DOI:** 10.3389/fnbeh.2019.00119

**Published:** 2019-06-06

**Authors:** Szabolcs Biró, Bálint Lasztóczi, Thomas Klausberger

**Affiliations:** Center for Brain Research, Division of Cognitive Neurobiology, Medical University of Vienna, Vienna, Austria

**Keywords:** cognitive flexibility, rule-switching, prefrontal cortex, virtual reality, behavioral task, optogenetics, head-fixed

## Abstract

Cognitive flexibility is the innate ability of the brain to change mental processes and to modify behavioral responses according to an ever-changing environment. As our brain has a limited capacity to process the information of our surroundings in any given moment, it uses sets as a strategy to aid neural processing systems. With assessing the capability of shifting between task sets, it is possible to test cognitive flexibility and executive functions. The most widely used neuropsychological task for the evaluation of these functions in humans is the Wisconsin Card Sorting Test (WCST), which requires the subject to alter response strategies and use previously irrelevant information to solve a problem. The test has proven clinical relevance, as poor performance has been reported in multiple neuropsychiatric conditions. Although, similar tasks have been used in pre-clinical rodent research, many are limited because of their manual-based testing procedures and their hardware attenuates neuronal recordings. We developed a two-choice rule-switch task whereby head-fixed C57BL/6 mice had to choose correctly one of the two virtual objects presented to retrieve a small water reward. The animals learnt to discriminate the visual cues and they successfully switched their strategies according to the related rules. We show that reaching successful performance after the rule changes required more trials in this task and that animals took more time to execute decisions when the two rules were in conflict. We used optogenetics to inhibit temporarily the medial prefrontal cortex (mPFC) during reward delivery and consumption, which significantly increased the number of trials needed to perform the second rule successfully (i.e., succeed in switching between rules), compared to control experiments. Furthermore, by assessing two types of error animals made after the rule switch, we show that interfering with the positive feedback integration, but leaving the negative feedback processing intact, does not influence the initial disengagement from the first rule, but impedes the maintenance of the newly acquired response set. These findings support the role of prefrontal networks in mice for cognitive flexibility, which is impaired during numerous neuropsychiatric diseases, such as schizophrenia and depression.

## Introduction

Cognitive flexibility is a crucial executive function which allows adaptive behavior by switching between different thoughts and actions, the complex rules of which are yet unknown. Deficit of this function has been observed in numerous neurological conditions, including schizophrenia, Alzheimer’s and Parkinson’s disease, autism spectrum disorder and unipolar depression (Downes et al., 1989; Freedman and Oscar-Berman, 1989; Hughes et al., 1994; Elliott et al., 1995; Merriam et al., 1999). Cognitive flexibility in humans has been measured with various behavioral methods, such as the Wisconsin Card Sorting Test (WCST; Berg, 1948) and the Cambridge Neuropsychological Test Automated Battery Intra-Extra Dimensional Set Shifting task (Robbins, 2000). These assessments measure rule acquisition and rule switching ability by a set of compound visual stimuli, with two or more superimposed perceptual dimensions. Subjects are required to categorize presented figures dependent upon their properties along these dimensions. The rule of discrimination itself is not explained to the participants; instead, feedback on the accuracy is provided after each response. Several trials after the initial rule acquisition, the sorting rule changes unbeknownst to the test subjects and the new rule has to be discerned. Many of the patients with the aforementioned conditions can resolve the initial rule for sorting or recognize the rule change, but because of perseveration of pre-potent responses, they have difficulties with adjusting their behavior once the relevance of categories changes.

Historically the WCST has been used to detect prefrontal damage in humans, signifying a critical role for prefrontal circuits in behavioral flexibility (Berg, 1948; Milner, 1963; Nelson, 1976; Lombardi et al., 1999). Human and non-human primate studies showed that the prefrontal cortex (PFC) coordinate multiple cognitive processes essential for shifting between rule-based strategies, including attentional set formation, rule encoding, and feedback integration amongst others (Monchi et al., 2001; Wallis et al., 2001; Lie et al., 2006; Mansouri et al., 2006; Reverberi et al., 2012). Although the anatomical homology of primate and rodent PFC is controversial (Preuss, 1995), a wealth of studies indicate that the rodent PFC might provide some cognitive capacities similar to primates. It has been demonstrated that activity patterns of neuronal populations in the medial PFC (mPFC) relate to abstract rules, behavioral responses, and conflicts of strategies during rule switching (Durstewitz et al., 2010; Bissonette and Roesch, 2015). In line with these observations, pharmacological inactivation or lesion of mPFC in rodents did not influence learning stimulus-response associations but hindered the application of new strategies upon rule contingency change (Ragozzino et al., 1999a,b; Birrell and Brown, 2000; Bissonette et al., 2008). These behavioral and functional similarities across species also proved rats and mice, to be compelling animal models in pre-clinical research of cognitive flexibility.

Even though components of such psychometric tests employed in human studies have been modified and adapted for rodent research, most tasks available to date still suffer some methodological limitations when it comes to fine dissection of neuronal circuits underlying cognitive flexibility (Bissonette et al., 2013). The most widely used set-shifting tasks made use of instinctive behavior and mimicking naturally occurring attentional sets, such as navigation, digging, taste and odor with great success (Birrell and Brown, 2000; Lagler et al., 2016; Malagon-Vina et al., 2018). On the other hand, they are manual based, requiring the experimenter to continuously interact with the test subject and the low trial number occasionally restricts statistical measures. A recent development of operant-based tasks, which combined automation with previously mentioned naturally occurring stimuli (odor, tactile, and visual), resolved this obstacle and it proved to be an effective tool in large scale pharmacological and genetic assessments (Scheggia et al., 2014). However, some difficulties still remain due to the chamber and the freely behaving design, which complicates neuronal recordings, in addition to the small and variable number of trials. These persisting limitations reveal an evident need for an automated task that allows a more sophisticated dissection of neuronal networks underlying cognitive flexibility by providing reliable measures and attenuating the difficulties of integrating cutting-edge recording techniques.

## Materials and Methods

### Experimental Subjects

All procedures were carried out under a license approved by the Austrian Ministry of Science and animals were tested in accordance with the regulations of the Medical University of Vienna. The test subjects were in-house bred adult male C57BL/6 mice (25–30 g), between 2 and 3 months of age. Before any experiments were carried out, the animals were housed two to eight per cage in a climate-controlled (temperature: 21°C ± 2°C, humidity: 50% ± 20%) animal facility, maintaining a 12-h, non-reversed light-dark cycle, starting at 7 am with *ad libitum* access to food and water. Procedures and tests were conducted during the light phase.

### Surgical Procedures

For the head-plate implantation, mice were anesthetised with isoflurane (4% induction, 2% maintenance) and their skull was fixed to a stereotactic frame, while the body temperature was stabilized with a heating pad. The skull was exposed, cleaned and sterilized with alcohol (70%) and iodine tincture, respectively. After the future craniotomy sites were marked [Bregma anterior-posterior (AP) 1.7 mm, medial-lateral (ML) ± 0.5 mm], the exposed skull was applied a coating of super glue to prevent bone infections. The stability of the head-plate was ensured with screws tightened into the nasal and the parietal bones, covered with acrylic cement (Refobacin^®^, Biomet). The exposed part of the skull was covered with silicon (Kwik-Sil, World Precision Instruments) to further attenuate the possibility of bone infection. Metamizol (Novalgin) was used as post-surgery analgesic. Following surgery, mice were housed individually. In addition, after the animals learned to perform the rule switch (conditions mentioned later), they underwent a craniotomy procedure in order to be able to collect electrophysiological data by inserting acute silicone probes into the mPFC. These data were not considered for the purpose of the current study and will be employed in another publication.

### Behavioral Training

**Figure 1:**
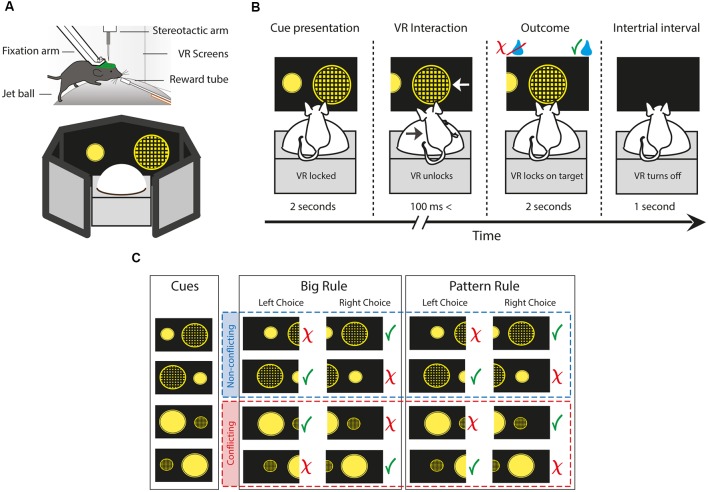
The behavioral setup and paradigm of the two choice rule-switching task. **(A)** Schematic illustrating a head-fixed mouse running on a styrene ball supported by constant air flow. The virtual environment is presented on a 270° surround screen. **(B)** Each trial is divided into four parts. First, the animal is presented two figures. During the cue presentation, the movement of the animal is not conveyed to the system. When the virtual reality (VR) unlocks, the animal is allowed to carry out decisions by steering the ball to the side of the figure of choice, which moves towards the middle of the screen. The animal is given feedback by the delivery or the lack of a water reward. The beginning of a new trial is indicated by the blackout of the screen. **(C)** Diagram showing all cue variations, trial types and possible outcomes depending on the rule and choice. Green check marks indicate correct, red ex marks indicate incorrect responses. Red and blue dash-lined boxes group the non-conflicting and conflicting trials, respectively. The paradigm uses two perceptual dimensions: size and pattern. The animal has to turn the ball towards the side of the figure which is either bigger or has a pattern on it, contingent on the rule.

After animals fully recovered from the implantation surgery, *ad libitum* water was taken away to start the water restriction. Mice were closely monitored to get ~1 ml water each day to avoid losing more than 15% of their body weight. After 2–3 days of habituation, mice were introduced to a PhenoSys virtual reality (VR) system (Figure 1A). The head-plate was tightened to the head-fixation arm and the orientation of the reward tube was set before each session, such that the water droplets touch the mouth of mouse upon delivery. The animals were given ~5 μl water every 7 s until satiety. After the animals learned to accept water droplets from the water delivery tube (2–3 days), they started the first level of the training process. In this stage, they learnt to interact with the VR through the JetBall and to discriminate figures upon their size. To achieve this, animals were trained twice daily for an hour (~600 trials per training session) with 7 h of difference, to choose the bigger from the presented figures by rolling the ball to the corresponding side. At early stages of training, if an incorrect choice was made, the animal had to re-do the same trial. The “insist” on correcting errors helped the mice learning the paradigm, as well as it prevented unwanted satiety before any progress was achieved. On the other hand, mice had to be closely monitored not to use “insist” as a strategy (in case of error, choosing the other side) instead of learning the rules to acquire reward. These strategies were diminished with behavioral shaping (taking “insist” out partially or fully). Once the performance reached a 90% success rate over 40 trials (in about 1 week), the training schedule was changed to one training session per day in the afternoon, which lasted 30–50 min, depending on the performance of the animal, corresponding to approximately 300–500 trials. Mice were then similarly trained to discriminate figures upon their face pattern (i.e., to form the “Pattern” set). After they achieved the same criterion of performance choosing the patterned figure as well (approximately 1 week), they had to follow the “Big” rule on the next training session, again until their performance reached 90% over a 40-trial period. Alternating “Pattern” and “Big” rules in different training sessions continued until the animals could execute both rules with high proficiency in different sessions, on two consecutive days (~8 weeks of training). Finally, the animals were introduced to the rule change within a session (i.e., rule-switch sessions). The rule-switch sessions were preceded by two training sessions on the initial rule, meaning, that the first rule of the rule-switch session was the same as the rule of the last two training sessions. The criterion of the successful rule acquisition was to achieve 13 correct trials in a 15-trial window (87% correct). The automatic rule switch was triggered when mice gave altogether 50 correct responses, where the last 14 were consecutively correct. As our behavioral paradigm was developed primarily as a tool to measure neuronal activity and the effects of neuronal manipulations on the behavior, the criterion for triggering the rule switch was designed to provide an extended period of good performance. Therefore, baseline data could be acquired in various conditions before the rule switch, which facilitates the detection of significant changes post-criterion.

### Rule-Switching Paradigm and Behavioral Analysis

During a single trial of the task, first, the VR turns on and the animal is presented with the cues (Figure 1B). During the cue presentation, the VR is locked for 2 s, meaning the movement of the mouse is not registered by the system. Hence, animals have sufficient time for decision making, and it also avoids unintentional choices by steering inaccuracy. Once the VR unlocks, the animal is allowed to carry out decisions by steering the ball to either side. The ball movement drives the figure of the corresponding side towards the middle of the screen. When the VR movement reaches 30° on either side, it locks onto the chosen figure. At the same time, the animal is given feedback by the delivery or the lack of a water droplet, dependent upon whether it was a correct or incorrect response, respectively. After 2 s the VR turns off for another second, indicating the beginning of a new trial. Although the system with the current settings is not well suited to pinpoint the exact start of the decision execution, this time period is an adequate temporal measure of response. Also, it is worth noting that the length of this episode is determined completely by the response time of the animal, ranging from 100 ms to 3 s. Hence, trials with decision execution times greater than 3 s were considered grooming periods and were excluded from the analysis.

After the rule switch, trials can be divided into two main types by the presented cues (Figure 1C). In non-conflicting trials, giving a correct response following either of the rules results in a correct response (e.g., left: big patterned circle vs. right: small plain circle). On the other hand, in conflicting trials, the two rules oppose each other, thus a correct response according to the previously reinforced rule results in an incorrect response choice (e.g., left: small patterned vs. right: big plain). Trial contingency was programmed to have a 60% bias towards conflicting trials to help acquisition of the new rule and to achieve more powerful analysis. Errors after the rule switch were categorized as perseverative and regressive types in conflicting trials, while nonsense types during non-conflicting trials. Perseverative errors were choices, where animals pursue the subsequent rule following a negative feedback but prior to the first correct response. This indicates the persistent use of the initial response set, despite the evidence of the relevant category change. Errors were marked regressive after the first correct conflicting trial, as animals “regressed” to the no longer reinforced rule. Thus, regressive errors demonstrate the unsuccessful maintenance of the new cognitive rule, notwithstanding the positive feedback of correct trials. Finally, nonsense errors were responses which following neither of the rules resulted in reward, hence they were never-reinforced (small plain circle). Behavioral data analysis was performed using standard functions and custom-made scripts in MATLAB (MathWorks).

### Optogenetic Procedures

Mice were anesthetized in the aforementioned way for the virus injection and optic fiber implantation. The AAV2/1-mDlx-channelrhodopsin (ChR2), an adeno-associated virus vector was bilaterally injected, that drives ChR2 expression through a mDlx enhancer, that restricts the expression of reporter genes to GABAergic cells. Specifics of the viral strategy for targeting and manipulating GABAergic interneurons were earlier described in detail (Dimidschstein et al., 2016). 0.5 μl virus was injected with the help of a pulled glass pipette into the prelimbic/infralimbic (PL/IL) area of the mPFC [Bregma AP 1.7 mm, ML, ± 0.3 mm, dorsal-ventral (DV) 1.5 mm] using a microsyringe pump. Two pieces of optic fiber (Ø200 μm, 0.22 NA, Thorlabs) were implanted transcranially above the PL area (1.7 mm AP, ± 0.4 mm ML, 1 mm DV) for bilateral stimulation. The position was secured by embedding the optic fibers in acrylic cement, firmly fixed to the head-plate. Behavioral experiments began 2–3 weeks after the virus injection.

After mice recovered from the optic fiber implantation surgery, they were trained for the rule-switching task the aforementioned way for 2–3 weeks, until the ChR2 protein was expressed in the target inhibitory cells. Experiments were scheduled in such a manner as two control and two optogenetic rule-switch sessions of each type (“Pattern to Big” and “Big to Pattern”) would follow each other. Mice were connected to the laser through a fiber optic cable. The head-plate and the optic fiber implant were covered with an opaque head-piece to avoid the laser light to interfere with the vision of the animal, in both testing and control conditions for comparability reasons. During initial optogenetic experiments, blue light (473 nm) was shined with a stimulation protocol (7 ms ON, 3 ms OFF) yielding an illumination intensity of 10–15 mW measured at the tip of the implanted fiber (*n* = 2). Subsequent optimization of the stimulation protocol (5 ms ON, 15 ms OFF) resulted in 0.1–1 mW power output (*n* = 2), which sufficiently silenced the mPFC as well. The laser was driven by digital computer signals (TTL pulses) of the PhenoSys system controlled by a custom-written MATLAB script on the controlling computer. The laser was shined in every rewarded trial during reward consumption and the inter-trial interval.

### Histology

After all behavioral and optogenetic experiments were finished, animals were anesthetized with urethane (3 g/kg) and intra-cardially perfused with saline followed by a fixative solution (4% paraformaldehyde, 0.05% glutaraldehyde, 15% saturated picric acid in 0.1 M phosphate buffer, pH ~7.4). The extracted brains were sectioned (coronal) with a vibratome (Leica VT 1000S, 70 μm thickness). Incubations and standard procedures used were described previously (Somogyi et al., 2004). ChR2 expression in the GABAergic cells was tested with double immunofluorescent reactions on individual free-floating sections with antibodies against ChR2 (mouse monoclonal; PROGEN Biotechnik GmbH; dilution: 1:10,000; for antibody specificity see Kleinlogel et al., 2011) and vesicular GABA transporter (guinea pig polyclonal; Frontier Institute Co., Ltd.; dilution: 1:500; for antibody specificity see Miyazaki et al., 2003; Fukudome et al., 2004) as detected with secondary antibodies conjugated to Alexa Fluor^®^ 488 or Cy^®^5 (Jackson ImmunoResearch Laboratories, Inc., West Grove, PA, USA) and imaged with immunofluorescent confocal microscopy [ZeissLSM 780; 63× oil immersion objective (NA 1.4)]. Positions of the optic fibers were assessed using a transmitted light microscope. One subject had to be removed from the optogenetic study for reasons of implant disposition.

All original data from this study will be made available upon reasonable request.

## Results

### Behavioral Performance

Mice (*n* = 7) were trained to discriminate two visual cues presented in a virtual environment and, by following rules, make decisions dependent upon different perceptual dimensions of size or pattern. After a brief cue presentation, the animals had to turn the ball to left or to the right, corresponding to the side of the chosen figure. For example, if the rule was “Pattern,” the target side was where the patterned figure was positioned. Upon a correct choice, animals collected water reward as a positive feedback, while in case of an incorrect choice, the negative feedback was the lack of reward. After the animals reached an extended period of stable good performance (50 correct responses, with the last 14 consecutively correct), the rule change was triggered. Following the previous example, if the starting rule was “Pattern,” the second rule was switched to “Big” and the animal had to turn the ball to the side where the bigger figure was positioned. In order to succeed afterward, animals had to recognize the rule change, disengage from the first rule, infer and apply the new strategy by attending to other attributes of the same visual cues. The paradigm of the task is described in the “Materials and Methods” section in detail.

**Figure 2:**
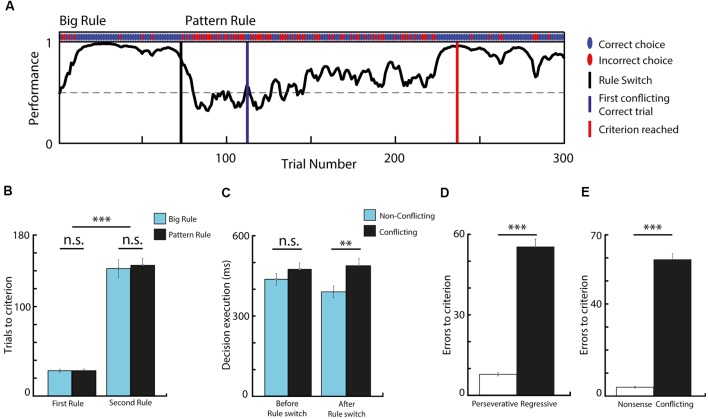
Task performance and behavioral analysis. **(A)** Performance curve deduced from the binary data (correct vs. incorrect choices) *via* Markov-chain Monte–Carlo analysis of one behavioral session. **(B)** Comparing the number of trials needed to reach the criterion before and after rule switch, in respect to rule modality. The second rule required significantly more trials to reach criterion, while the rule-type had no effect. **(C)** Data showing decision execution time before and after rule switch, during conflicting and non-conflicting trials. Animals spent significantly more time making choices during conflicting trials compared to non-conflicting after the rule switch. **(D)** Bar graph comparing the number of errors before and after the first conflicting correct trial. Animals made significantly more regressive than perseverative errors. **(E)** Data showing that very small number of nonsense errors were made. *n* = 7 animals, **p < 0.01; ***p < 0.001; n.s. not significant; error bars show SEM.

All mice trained to perform the set-shifting task managed to successfully discriminate between the visual cues and reached the criteria for successful performance during both the first and the second rule (Figure 2A). Altogether, 145 rule-switch session (out of 169) were considered successful, where 76 were of “Big-to-Pattern” and 69 were of “Pattern-to-Big” type. The analysis of the behavioral performance revealed that significantly more trials were needed to reach the criterion for the second rule, than for the first rule (*t*-test, means 28.3 vs. 144.4, SEM 1.4 and 14.8, *p* < 0.001, *t* = −17.8, *df* = 288, Cohen’s *d* = −2.09, effect size = −0.72). To examine the effect of rule-shift and the visual cues on trial number needed to reach the criterion, a two-way ANOVA was conducted (Figure 2B). The simple main effects analysis showed that while switching from the first rule to the second rule increased the number of trials needed to reach the criterion (*F*_(1,24)_ = 185.78; *p* < 0.001), the rules *per se* (whether it was big or pattern) did not influence it (*F*_(1,24)_ = 0.19; *p* = 0.668), neither did the two factors interact (*F*_(1,24)_ = 0.13, *p* = 0.7167). Comparing trial length in different conditions (Figure 2C) revealed a significant difference between non-conflicting and conflicting trials after the rule switch (*t*-test, means 0.394. vs. 0.489, respectively; SEM 0.022 and 0.027, respectively; *p* = 0.007; *t* = 2.7292, *df* = 288, Cohen’s *d* = −0.32, effect size = −0.16). This indicates that on trials where the two rules were in conflict, the animals took more time carrying out decisions. Altogether, these data confirm that animals learned to discriminate the presented cues, followed the appropriate rule, and they had difficulty switching between them when the relevant category changed.

As errors during conflicting trials after the rule switch provide essential feedback for cognitive rule adjustment, we categorized set-shifting errors as perseverative and regressive. During perseverative responses, subjects fail to shift to a new response set despite the negative feedback, and they execute choices following the previous rule, which does not apply anymore. Errors become regressive after subjects make the first conflicting correct choice, indicating that the newly-reinforced response set is identified, but then they are unable to maintain it, and instead they revert back to choices in accordance with the initial rule. Analyzing these two types of errors (Figure 2D) concluded that most of the errors were of regressive-types (*t*-test, means 7.75 vs. 55.35, SEM 0.62 and 2.92, *p* < 0.001, *t* = −15.9, *df* = 266, Cohen’s *d* = −1.95, effect size = −0.7), which implies that mice had difficulty suppressing responses to the initial set. Additionally, animals made very few “non-sense” errors (i.e., choices that were never reinforced; Figure 2E; *t*-test, means 59.22 vs. 3.87, SEM 2.67 and 0.44, *p* < 0.001, *t* = 20.5, *df* = 266, Cohen’s *d* = 2.5, effect size = 0.78), which suggests that other rule possibilities were less likely to be explored and that the mice acquired a high ball-handling precision during the training, making very few mistakes as a result of steering inaccuracy.

### Optogenetic Experiments

**Figure 3:**
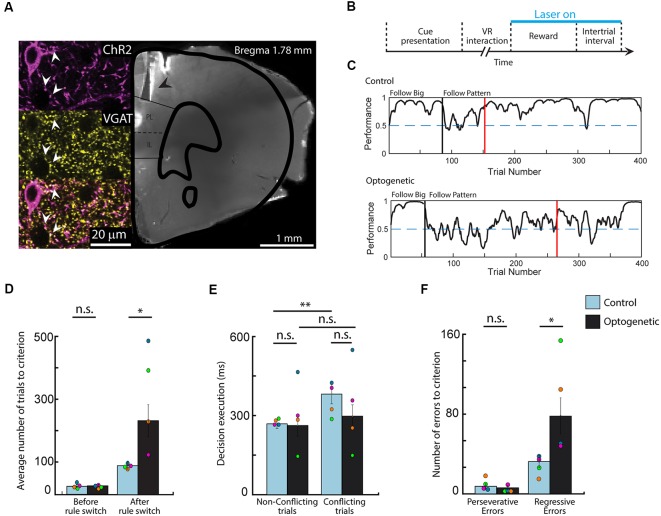
Optogenetic silencing of the medial prefrontal cortex (mPFC) during positive feedback epochs induces symptoms of cognitive rigidity. **(A)** Photomicrograph and confocal scan of a brain slice indicating the location of the optic fibers (black arrow) and the immunolabeling of channelrhodopsin-2 (ChR2) expression in GABAergic cells in the prelimbic/infralimbic (PL/IL) area (white arrow). **(B)** Diagram of the optogenetic stimulation protocol. Light was shined in every rewarded trial, before and after rule switch (7 ms ON, 3 ms OFF; 10–15 mW; *n* = 2 and 5 ms ON, 15 ms OFF; 0.1–1 mW; *n* = 2). **(C)** Behavioral curves showing performance difference in rule switching during one control and one optogenetic session from the same animal. Performance was assessed using the binary data of correct and incorrect choices *via* Markov-chain Monte–Carlo analysis. Black vertical lines indicate the automated rule switch, while the red vertical lines mark the beginning of the 13 out of 15 correct trials. **(D)** Number of trials needed to reach the criterion on the first and on the second rule. **(E)** Comparing decision execution times after the rule switch shows that silencing the mPFC diminished the difference between conflicting and non-conflicting trial length in optogenetic experiments. **(F)** Data showing increased number of regressive errors but not perseverative errors during optogenetic sessions. *n* = 4 animals, *p < 0.05; **p < 0.01; n.s. not significant; error bars show SEM; Different colors mark the individual animals’ averages.

To determine the behavioral effect of silencing the mPFC during positive-feedback epochs, we implemented an optogenetic system (Passecker et al., 2019), which achieves locally restricted inhibition of principal neurons through activation of GABAergic interneurons. For these experiments the same animals (*n* = 4), which underwent the earlier described behavioral tests, were bilaterally injected with an AAV2/1-mDlx-ChR2 virus (Dimidschstein et al., 2016), to selectively express the light-sensitive ChR2 channel in GABAergic cells (Figure 3A). Optic fibers were implanted above the PL area of the mPFC. After the animals recovered and their behavioral performance returned to a pre-surgery level, the task was performed in alternating sessions, with and without optogenetic stimulation, granting the advantage of comparing optogenetic and control experiments within the same animal. Inhibitory cells were activated by light application during reward delivery and the inter-trial interval in all rewarded trials, before and after rule switch (Figure 3B). As expected, performance on the initial rule was not affected by inhibition of the mPFC (Figure 3C). Mice took the same number of trials to reach the criterion, in both type of sessions (Wilcoxon rank sum, means 24.50 vs. 24.30, SEM 3.69 and 3.25, *p* = 0.82, *z* = 0.23). In contrast, reaching the criterion after a rule switch to the second rule took significantly more trials during light on, compared to control sessions (Wilcoxon rank sum, means 235.6 vs. 92.5, respectively; SEM 50.65 and 12.76, respectively; *p* = 0.0111, *z* = 2.54), which highlights an important role for the mPFC in reward integration during set-shifting, but not during single rule performance (Figure 3D).

To test whether optogenetic inactivation of mPFC had any effect on decision making and decision execution, we analyzed the lengths of VR interaction times of various trial conditions after the rule switch (Figure 3E). Similarly to the full dataset (Figure 2C), in control sessions, virus injected animals took more time to respond to the presented cues when the rules were in conflict (*t*-test, means 0.268 vs. 0.381, SEM 0.18 and 0.37, *p* = 0.002, Cohen’s *d* = −1.13, effect size = −0.49), while this difference was not observed in light on sessions (*t*-test, means 0.283 vs. 0.326, SEM 0.41 and 0.48, *p* = 0.123, Cohen’s *d* = −0.28, effect size = −0.14), suggesting that animals had difficulties suppressing impulsive responses when the normal activity of the mPFC is disturbed. However, decisive conclusions cannot be drawn as neither the conflicting (Wilcoxon rank sum, means 0.381 vs. 0.327, SEM 0.37 and 0.48, *p* = 0.3734, *z* = −0.89) nor the non-conflicting trial lengths (Wilcoxon rank sum, means 0.268 vs. 0.283, SEM 0.18 and 0.41, *p* = 0.9737, *z* = −0.03) differed in the two experimental settings when compared to each other. Lastly, assessing the number of perseverative and regressive errors (Figure 3F) showed that mice made markedly more regressive errors during optogenetic experiments compared to control sessions (Wilcoxon rank sum, means 78.30 vs. 32.25, respectively; SEM 18.28 and 6.00, respectively; *p* = 0.038, *z* = −2.08), while the number of perseverative errors did not differ in the two session types (Wilcoxon rank sum, means 6.10 vs. 6.92, respectively; SEM 1.87 and 1.77, respectively; *p* = 0.8685, *z* = −0.17). These results imply, that interfering with post-reward computations in the mPFC has no effect on the ability to alter cognitive rules and responses, though it hinders the maintenance of newly acquired response sets causing mice regressing more to no longer reinforced choices.

## Discussion

Technological developments of recent years have triggered an interest in implementing and updating mouse behavioral tasks to more reliably measure cognitive functions (Cho et al., 2015; Havenith et al., 2018; Pinto et al., 2018). Their genetic flexibility, commercial availability, and their tolerance of head-fixation appointed them as an ideal animal model for experimental neuroscience (Trancikova et al., 2011; White, 2016; Stowers et al., 2017; Chen et al., 2019). In this study, we introduce a visual two-choice rule-switching task developed for head-fixed mice, which opens up new possibilities in preclinical research of cognitive flexibility. In this paradigm, mice learned to execute choices with high precision through an air-supported ball connected to a VR system to follow abstract rules contingent upon the size and the pattern of the presented cues. The trial length was designed to be short, which resulted in a large number of trials, allowing robust statistical measures and decreased variance. Our data shows that mice learned to differentiate the visual cues and they were able to switch between the appropriate strategies without having bias towards any of the task sets. Using bivalent visual cues effectively increased the number of trials needed to establish a good performance after the rule switch, which yielded several thousands of trials cumulatively. The performance decrement after rule switch also indicates that mice faced difficulty reconfiguring task-sets. This switch cost was also indicated by the increased decision making times on trials where rules were in conflict. These phenomena are well described in human rule switching (Wylie and Allport, 2000; Monsell, 2003; Schneider and Logan, 2007) and are in agreement with data from previous studies in mice showing that response time increases when difficult choices are made (Abraham et al., 2004; Young et al., 2010).

We also explored the behavioral effect of disrupting reward integration in the mPFC on switching between tasks by optogenetically silencing the area during post-reward epochs. Our optogenetic experiments had some limitations regarding the low subject number, which introduced higher variance, and it is missing the viral control to test whether the laser light, *per se*, had any effect on the behavior. However, our results show no significant difference between the optogenetic and the control sessions during the initial rule acquisition, while the performance in sessions with optogenetic silencing of mPFC is clearly poorer after the rule switch. This finding suggests that the light itself did not influence the behavior, as it aligns well with previous research showing that disruption of the mPFC does not affect the initial rule performance in a task switch paradigm, it only hinders the acquisition of the second rule (Hampshire and Owen, 2006; Bissonette et al., 2008; Keeler and Robbins, 2011).

The mPFC is theorized to govern multiple cognitive processes, which work together to achieve a successful set shifting after rule change. These include initiation of new choices, inhibition of subsequent, ineffective responses, and promoting newly acquired, effective strategies. These processes can be monitored by delineating errors as perseverative and regressive types (Ragozzino, 2007; Gastambide et al., 2012). Human, non-human primate, and rodent studies suggest that mPFC is involved in the initial suppression of established response sets after rule contingencies change, thus promoting the selection of new choices, marked by an increase in perseverative errors (Dias et al., 1997; Ragozzino et al., 2003; Chudasama and Robbins, 2006; Ragozzino, 2007). In contrast, an increase in regressive errors indicates a failure to maintain the newly acquired response sets, which is dependent on the basal ganglia (Ragozzino et al., 2002; Ragozzino and Choi, 2004; Floresco et al., 2006; Palencia and Ragozzino, 2006). Thus, the cooperation of these functionally different brain areas facilitates cognitive flexibility by choosing an alternative response and promoting it over other possibilities. Contrary to earlier findings, however, we found that silencing the mPFC increased the number of regressive errors, instead of the perseverative errors. A possible explanation for these results may be the way perseverative errors were defined in the previous rodent studies. In these experiments, a window of multiple trials was applied and perseveration was defined when the majority of the trials in that block were incorrect. This definition of perseverative errors includes both perseverative and regressive errors, as defined here, and it does not quite capture the initial shift in response set, when the animal disengages from the primary rule for the first time. Furthermore, Oualian and Gisquet-Verrier who observed some perseverative behavior in their lesion studies as well, argue that in most of the aforementioned experiments animals chose to maintain the initial strategy because it still leads to reward for half of the trials (Delatour and Gisquet-Verrier, 1999, 2000; Oualian and Gisquet-Verrier, 2010). Following the previous rule after the rule change in our paradigm is a very inefficient strategy because of the small reward size, the length of the experiment, and the probability of encountering non-conflicting trials, which is only 40% after the rule change. Also, they hypothesized that the behavioral rigidity caused by the dysfunctional mPFC is due to the attenuated ability to resolve internal conflicts generated by the opposing previously learned strategy and the new rule. Therefore, our findings imply a more complex cooperation between the mPFC and the dorsomedial striatum in behavioral flexibility, suggesting that processing only negative feedback signals in the mPFC is enough to initiate a new response after rule contingencies change, but to successfully maintain it over the subsequent rule, positive feedback signals of the striatum have to be integrated in the mPFC.

As cognitive rigidity is prevalent in a large number of psychiatric disorders, continuous development of preclinical research tools is essential in order to dissect and understand the complex mechanisms, which bring about flexible behavior. The system we developed is well suited for neuronal recordings, as head-fixation augments stability and it has further advantages combined with other movement-sensitive techniques such as juxta-cellular recording and labeling (Lapray et al., 2012; Kőszeghy et al., 2018). It is completely automatic, including sampling and synchronization of behavioral and physiological data, which immensely simplifies data collection and data analysis. Its parameters are flexibly programmable to test other components of cognitive flexibility, such as attentional set-shifting and reversal learning. As the reliable assessment of set-shifting using visual based tasks remains a challenge in mouse research (Floresco et al., 2008), we would like to test whether our system is capable of measuring visual attentional set shifts as a next step. By introducing a novel shape of different sizes and patterns, a total change paradigm can be established (Slamecka, 1968), creating a task which is analogous to the ones used in clinical practice (Sahakian and Owen, 1992). Finally, as one of the goals of the development was to create a task for mice, implementing genetically modified mouse models of neurocognitive diseases which produce cognitive rigidity as a symptom, could give us a more detailed insight into the pathophysiology of these conditions. Thus, this adaptability of the apparatus provides a multifaceted approach to tackle behavioral and neurobiological questions of cognitive flexibility that were rather troublesome with previous tasks.

In conclusion, in this study, we demonstrate a cognitive flexibility task that integrates recent technological advancements of neuroscience to overcome limitations of currently used tasks. The head-fixed design provides stability for various neuronal recording techniques while using mouse models widens the horizon of preclinical research of psychiatric diseases. The abstract task sets prevent mice to develop biases towards choices and also increase the difficulty of solving the task, which combined with short trial length not only increases the available time interval for measurements but also boosts the statistical power. Our initial finding suggests that selectively silencing the mPFC during correct trials concurrently with reward consumption does not affect the initial rule performance, but it induces signs of cognitive rigidity when new rule strategies are implemented. Furthermore, our data indicate that interfering with post reward computations in the mPFC but leaving the negative feedback periods intact, hinders the maintenance of the new response set after rule switching, but it does not disturb the ability to initially disengage from the first task, suggesting the importance of mPFC for cognitive flexibility.

## Ethics Statement

This study was carried out in accordance with the recommendations of Austrian Ministry of Science. The protocol was approved by the Austrian Ministry of Science.

## Author Contributions

SB, BL and TK contributed to the conception and design of the study. SB carried out the experiments and performed the analysis of the data. SB and TK wrote the manuscript.

## Conflict of Interest Statement

The authors declare that the research was conducted in the absence of any commercial or financial relationships that could be construed as a potential conflict of interest.
